# Poly[[diaqua­(μ_4_-l-tartrato)(μ_2_-l-tartrato)dizinc(II)] tetra­hydrate]

**DOI:** 10.1107/S1600536810007543

**Published:** 2010-03-06

**Authors:** Hou-Ting Liu, Jing Lu, Da-Qi Wang

**Affiliations:** a School of Chemistry and Chemical Engineering, Liaocheng University, Liaocheng 252059, People’s Republic of China

## Abstract

In the title compound, {[Zn(C_4_H_4_O_6_)(H_2_O)]·2H_2_O}_*n*_, the l-tartrate ligands adopt μ_4_- and μ_2_-coordination modes. The Zn^II^ atom adopts an octa­hedral geometry and is chelated by two kinds of l-tartrate ligands through the hydr­oxy and carboxyl­ate groups and coordinated by one unchelating carboxyl­ate O atom and one water mol­ecule. In the crystal, the l-tartrate ligands link the Zn^II^ atoms, forming a two-dimensional coordination layer; these layers are futher linked into a three-dimensional supra­molecular network by O—H⋯O hydrogen bonds between the two-dimensional coordin­ation layers and the uncoordinated water mol­ecules. The latter are equally disordered over two positions.

## Related literature

For the potential applications and varied architectures and topologies of chiral inorganic–organic materials, see: Ma *et al.* (2007 ▶); Kitagawa *et al.* (2004 ▶); Lee *et al.* (2002 ▶). For chiral multifunctional materials constructed from tartrate, see: Liu *et al.* (2008 ▶) Gelbrich *et al.* (2006 ▶). For magnetic properties of transition metal tartrates, see: Coronado *et al.* (2006 ▶).
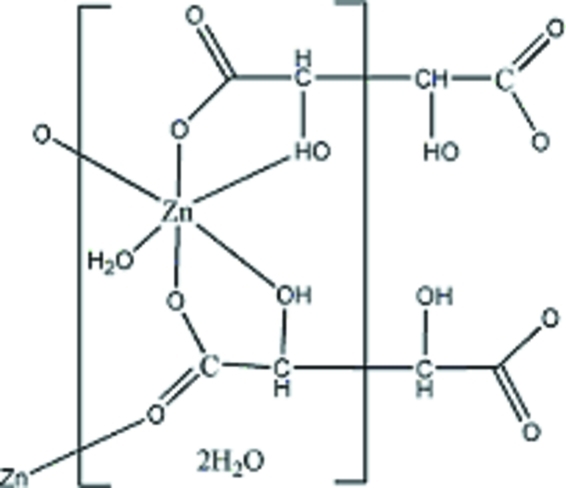


## Experimental

### 

#### Crystal data

[Zn(C_4_H_4_O_6_)(H_2_O)]·2H_2_O
*M* *_r_* = 267.49
Monoclinic, 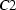

*a* = 12.8652 (16) Å
*b* = 8.7884 (14) Å
*c* = 8.3816 (12) Å
β = 114.130 (1)°
*V* = 864.9 (2) Å^3^
*Z* = 4
Mo *K*α radiation
μ = 2.87 mm^−1^
*T* = 296 K
0.50 × 0.48 × 0.45 mm

#### Data collection

Bruker SMART CCD area-detector diffractometer
Absorption correction: multi-scan (*SADABS*; Sheldrick, 1996 ▶) *T*_min_ = 0.328, *T*_max_ = 0.358
2182 measured reflections
1296 independent reflections
1262 reflections with *I* > 2σ(*I*)
*R*_int_ = 0.017

#### Refinement

*R*[*F* ^2^ > 2σ(*F* ^2^)] = 0.025
*wR*(*F* ^2^) = 0.071
*S* = 1.10
1296 reflections
147 parameters
1 restraint
H-atom parameters constrained
Δρ_max_ = 0.34 e Å^−3^
Δρ_min_ = −0.42 e Å^−3^
Absolute structure: Flack (1983 ▶), 481 Friedel pairs
Flack parameter: 0.01 (2)

### 

Data collection: *SMART* (Sheldrick, 2008 ▶); cell refinement: *SAINT* (Sheldrick, 2008 ▶); data reduction: *SAINT*; program(s) used to solve structure: *SHELXS97* (Sheldrick, 2008 ▶); program(s) used to refine structure: *SHELXL97* (Sheldrick, 2008 ▶); molecular graphics: *SHELXTL* (Sheldrick, 2008 ▶); software used to prepare material for publication: *SHELXL97*.

## Supplementary Material

Crystal structure: contains datablocks global, I. DOI: 10.1107/S1600536810007543/bq2195sup1.cif

Structure factors: contains datablocks I. DOI: 10.1107/S1600536810007543/bq2195Isup2.hkl

Additional supplementary materials:  crystallographic information; 3D view; checkCIF report

## Figures and Tables

**Table 1:** Hydrogen-bond geometry (Å, °)

*D*—H⋯*A*	*D*—H	H⋯*A*	*D*⋯*A*	*D*—H⋯*A*
O8—H8*D*⋯O2^i^	0.85	2.23	3.074 (10)	174
O9—H9*A*⋯O1^i^	0.85	2.41	2.849 (7)	113
O9—H9*A*⋯O7^i^	0.85	2.38	3.094 (8)	142
O9—H9*C*⋯O9^ii^	0.85	1.95	2.421 (14)	113
O8—H8*A*⋯O8′^ii^	0.85	2.16	2.791 (12)	131
O9—H9*C*⋯O9′^ii^	0.85	2.00	2.761 (10)	149
O9′—H9′*C*⋯O1^i^	0.85	1.92	2.765 (7)	177
O9′—H9′*C*⋯O2^i^	0.85	2.66	3.225 (8)	125

Symmetry codes: (i) 
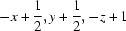
; (ii) 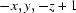.

**Fig. 1.**
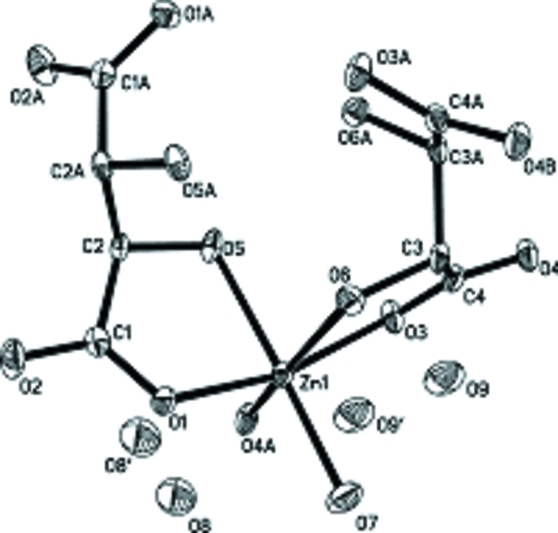
The molecular structure of (I) with 30% probability displacement elliposolids (hydrogen atoms are omitted for clarity). Symmetry codes: (A) 0.5-x, -0.5+y, -z; (B) -x, -2+y, -z.

**Fig. 2.**
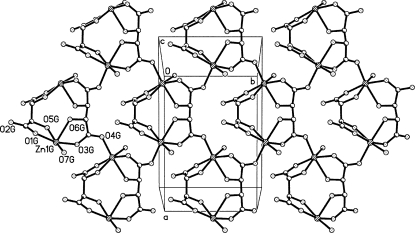
The two-dimensional structure of the title compound, with atom labels.

**Fig. 3.**
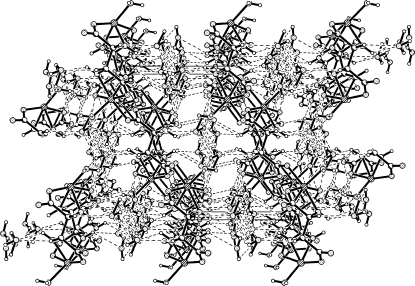
The three-dimensional supramolecular layer construcuted by hydrogen bonds.

## supplementary crystallographic information

### Comment

Chiral inorganic-organic materials have received much attention, not only because of their numerous potential applications in nonlinear optics, enantioselective catalysis and medicine, but also owing to their intriguing variety of architectures and topologies (Ma *et al.*, 2007; Kitagawa *et al.*, 2004; Lee *et al.*, 2002). L-tartaric acid, a simple and inexpensive chiral ligand source, was often used to construct novel chiral multifunctional materials (Liu *et al.*, 2008; Gelbrich *et al.*, 2006). Firstly, tartaric acid is flexible dicarboxylate ligands with two hydroxyl groups, and can offer more coordination sites and allow the formation of five- or six-membered ring, which can stabilize the solid network. Secondly, the deprotonated carboxylate group possesses polarizable system, it can transfer electrons easily. So, tartrate ligand is a good candidate of constructing chiral magnetic and chiral optical materials. In this paper, we reported the structure of the title compound, which is constructed by the chiral L-tartrate ligand.

X-ray single crystal diffraction studies reveal that the crystallographic unique unit of (I) is composed of one Zn^II^ ion, two halves of L-tartrate ligand, one coordination water and two disordered lattice water molecules with occupancies both in the 0.5:0.5 ratio. As shown in Fig. 1, two kinds of L-tart ligands chelate two Zn centers through the hydroxyl and carboxylate groups in cis confirmation to form [Zn_2_(L-tart)_2_] dimmer, which is similar to the reported tartrate salts (Coronado *et al.*, 2006). And, the octahedral geometry of Zn^II^ is completed by one unchelating carboxylate oxygen atom and one water molecule. For compound (I), L-tartrate ligands adopt µ_4_- and µ_2_- two coordination modes, which link the [Zn_2_(L-tart)_2_] dimmers to form two-dimensional coordination layer. The coordination and lattice water molecules hydrogen bond to the hydroxy and carboxylate groups, so the two-dimensional coordination layers are further linked together to form three-dimensional supramolecular network (shown in Fig. 2). The parameters of hydrogen bonds are listed in Table 1.

### Experimental

Compound (I) was obtained at room temperature. An aqueous solution (5 ml) of L-tartaric acid (0.51 g, 3.4 mmol) was added dropwise into an aqueous solution (10 ml) of Zn(OAc)_2_˙2H_2_O (0.37 g, 1.7 mmol). White crystals were obtained in yield about 60% (based on Zn) after the solution was allowed to stand for several days. Elemental analysis, Found: C 17.36, H 3.23%. Calc. for C_4_H_10_O_9_Zn : calcd. C 17.94, H 3.74%.

### Refinement

All H atoms were positioned geometrically and treated as riding on their parent atoms, with C–H 0.980, *O*(aqua)–H 0.850, *O*(hydroxyl)–H 0.970 and with *U*_iso_(H) = 1.2*U*_eq_(C). The O8 and O9 atom is resolved into two positions by PART instructions. The geometries and anisotropic displacement parameters of disordered atoms were refined with soft restraints using the SHELXL commands damp.

### Crystal data

**Table:**

[Zn(C_4_H_4_O_6_)(H_2_O)]·2H_2_O	*F*(000) = 544
*M**_r_* = 267.49	*D*_x_ = 2.054 Mg m^−^^3^
Monoclinic, *C*2	Mo *K*α radiation, λ = 0.71073 Å
Hall symbol: C 2y	Cell parameters from 1296 reflections
*a* = 12.8652 (16) Å	θ = 2.7–25.0°
*b* = 8.7884 (14) Å	µ = 2.87 mm^−^^1^
*c* = 8.3816 (12) Å	*T* = 296 K
β = 114.130 (1)°	Block, white
*V* = 864.9 (2) Å^3^	0.50 × 0.48 × 0.45 mm
*Z* = 4	

### Data collection

**Table:**

Bruker SMART CCD area-detector diffractometer	1296 independent reflections
Radiation source: fine-focus sealed tube	1262 reflections with *I* > 2σ(*I*)
graphite	*R*_int_ = 0.017
phi and ω scans	θ_max_ = 25.0°, θ_min_ = 2.7°
Absorption correction: multi-scan (*SADABS*; Sheldrick, 1996)	*h* = −15→14
*T*_min_ = 0.328, *T*_max_ = 0.358	*k* = −8→10
2182 measured reflections	*l* = −9→9

### Refinement

**Table:**

Refinement on *F*^2^	Secondary atom site location: difference Fourier map
Least-squares matrix: full	Hydrogen site location: inferred from neighbouring sites
*R*[*F*^2^ > 2σ(*F*^2^)] = 0.025	H-atom parameters constrained
*wR*(*F*^2^) = 0.071	*w* = 1/[σ^2^(*F*_o_^2^) + (0.0392*P*)^2^ + 0.821*P*] where *P* = (*F*_o_^2^ + 2*F*_c_^2^)/3
*S* = 1.10	(Δ/σ)_max_ = 0.001
1296 reflections	Δρ_max_ = 0.34 e Å^−^^3^
147 parameters	Δρ_min_ = −0.42 e Å^−^^3^
1 restraint	Absolute structure: Flack (1983), 481 Friedel pairs
Primary atom site location: structure-invariant direct methods	Flack parameter: 0.01 (2)

### Special details

**Table:**

Geometry. All esds (except the esd in the dihedral angle between two l.s. planes) are estimated using the full covariance matrix. The cell esds are taken into account individually in the estimation of esds in distances, angles and torsion angles; correlations between esds in cell parameters are only used when they are defined by crystal symmetry. An approximate (isotropic) treatment of cell esds is used for estimating esds involving l.s. planes.
Refinement. Refinement of *F*^2^ against ALL reflections. The weighted *R*-factor wR and goodness of fit *S* are based on *F*^2^, conventional *R*-factors *R* are based on *F*, with *F* set to zero for negative *F*^2^. The threshold expression of *F*^2^ > σ(*F*^2^) is used only for calculating *R*-factors(gt) etc. and is not relevant to the choice of reflections for refinement. *R*-factors based on *F*^2^ are statistically about twice as large as those based on *F*, and *R*- factors based on ALL data will be even larger.

### Fractional atomic coordinates and isotropic or equivalent isotropic displacement parameters (Å^2^)

**Table:**

	*x*	*y*	*z*	*U*_iso_*/*U*_eq_	Occ. (<1)
Zn1	0.23251 (3)	0.52758 (7)	0.15818 (5)	0.02164 (15)	
O1	0.2031 (2)	0.3568 (4)	0.2954 (4)	0.0271 (7)	
O2	0.1296 (3)	0.1272 (4)	0.2844 (4)	0.0418 (9)	
O3	0.2105 (2)	0.7016 (4)	−0.0093 (4)	0.0289 (7)	
O4	0.1403 (2)	0.9327 (3)	−0.1022 (4)	0.0307 (7)	
O5	0.0911 (2)	0.4067 (3)	−0.0441 (4)	0.0270 (7)	
H5	0.0531	0.4421	−0.1579	0.032*	
O6	0.0846 (2)	0.6635 (4)	0.1705 (4)	0.0265 (7)	
H6	0.0438	0.6340	0.2349	0.032*	
O7	0.3436 (3)	0.6376 (5)	0.3781 (4)	0.0447 (9)	
H7B	0.3339	0.6097	0.4681	0.054*	
H7C	0.3352	0.7335	0.3666	0.054*	
O8	0.1503 (7)	0.4516 (9)	0.6526 (9)	0.042 (3)	0.499 (12)
H8A	0.0955	0.4991	0.5745	0.050*	0.499 (12)
H8D	0.2121	0.5007	0.6784	0.050*	0.499 (12)
O9	0.0715 (6)	0.8274 (9)	0.4409 (8)	0.038 (2)	0.495 (11)
H9A	0.0955	0.8838	0.5312	0.057*	0.495 (11)
H9C	0.0299	0.7572	0.4531	0.057*	0.495 (11)
O8'	0.0611 (7)	0.4189 (9)	0.6283 (8)	0.042 (3)	0.501 (12)
H8'A	−0.0075	0.3939	0.5660	0.050*	0.501 (12)
H8'D	0.0751	0.5059	0.5974	0.050*	0.501 (12)
O9'	0.1034 (6)	0.6981 (9)	0.4957 (8)	0.038 (2)	0.505 (11)
H9'A	0.0453	0.7453	0.4937	0.045*	0.505 (11)
H9'C	0.1636	0.7473	0.5563	0.045*	0.505 (11)
C1	0.1372 (3)	0.2489 (5)	0.2135 (6)	0.0242 (9)	
C2	0.0635 (4)	0.2672 (5)	0.0178 (6)	0.0213 (10)	
H2	0.0786	0.1821	−0.0453	0.026*	
C3	0.0619 (4)	0.8000 (5)	0.0673 (6)	0.0240 (11)	
H3	0.0737	0.8879	0.1447	0.029*	
C4	0.1442 (3)	0.8113 (5)	−0.0218 (6)	0.0233 (9)	

### Atomic displacement parameters (Å^2^)

**Table:**

	*U*^11^	*U*^22^	*U*^33^	*U*^12^	*U*^13^	*U*^23^
Zn1	0.0224 (2)	0.0192 (2)	0.0234 (2)	−0.0043 (2)	0.00947 (16)	0.0007 (2)
O1	0.0278 (14)	0.0253 (17)	0.0275 (16)	−0.0077 (13)	0.0104 (12)	0.0035 (14)
O2	0.070 (2)	0.026 (2)	0.0321 (18)	−0.0124 (17)	0.0231 (17)	0.0034 (15)
O3	0.0307 (15)	0.0190 (16)	0.0466 (19)	0.0052 (13)	0.0257 (14)	0.0091 (14)
O4	0.0287 (14)	0.0182 (18)	0.0502 (19)	0.0017 (13)	0.0212 (14)	0.0064 (15)
O5	0.0375 (15)	0.0179 (17)	0.0333 (17)	−0.0090 (13)	0.0224 (13)	−0.0027 (13)
O6	0.0287 (14)	0.0259 (17)	0.0260 (15)	0.0033 (12)	0.0124 (12)	−0.0001 (12)
O7	0.051 (2)	0.048 (2)	0.0279 (16)	−0.0214 (18)	0.0087 (15)	−0.0063 (17)
O8	0.050 (6)	0.041 (5)	0.030 (4)	−0.001 (3)	0.012 (3)	0.002 (3)
O9	0.043 (4)	0.044 (6)	0.023 (4)	−0.007 (3)	0.010 (3)	−0.007 (3)
O8'	0.050 (5)	0.041 (4)	0.029 (4)	−0.001 (4)	0.012 (3)	0.002 (3)
O9'	0.043 (4)	0.044 (5)	0.023 (4)	−0.007 (3)	0.010 (3)	−0.008 (3)
C1	0.0273 (19)	0.023 (2)	0.027 (2)	0.0014 (18)	0.0157 (17)	0.0015 (18)
C2	0.028 (2)	0.014 (2)	0.026 (2)	−0.0046 (17)	0.0149 (18)	0.0000 (18)
C3	0.028 (2)	0.012 (2)	0.036 (3)	−0.0012 (18)	0.017 (2)	−0.0045 (19)
C4	0.0201 (18)	0.017 (2)	0.034 (2)	−0.0032 (15)	0.0125 (17)	−0.0028 (18)

### Geometric parameters (Å, °)

**Table:**

Zn1—O3	2.016 (3)	O8—H8A	0.8501
Zn1—O1	2.019 (3)	O8—H8D	0.8500
Zn1—O4^i^	2.054 (3)	O8—H8'D	1.0056
Zn1—O7	2.054 (3)	O9—H9A	0.8500
Zn1—O5	2.189 (3)	O9—H9C	0.8500
Zn1—O6	2.285 (3)	O9—H9'A	0.9764
O1—C1	1.270 (6)	O8'—H8A	1.0296
O2—C1	1.247 (6)	O8'—H8'A	0.8500
O3—C4	1.264 (5)	O8'—H8'D	0.8500
O4—C4	1.252 (5)	O9'—H9'A	0.8500
O4—Zn1^ii^	2.054 (3)	O9'—H9'C	0.8500
O5—C2	1.431 (5)	C1—C2	1.531 (6)
O5—H5	0.9300	C2—C2^iii^	1.537 (9)
O6—C3	1.437 (6)	C2—H2	0.9800
O6—H6	0.9300	C3—C4	1.529 (6)
O7—H7B	0.8500	C3—C3^iii^	1.529 (10)
O7—H7C	0.8500	C3—H3	0.9800
			
O3—Zn1—O1	162.78 (11)	H8D—O8—H8'D	120.0
O3—Zn1—O4^i^	92.67 (13)	H9A—O9—H9C	109.5
O1—Zn1—O4^i^	100.41 (13)	H9A—O9—H9'A	95.4
O3—Zn1—O7	96.64 (15)	H9A—O9—H9'C	77.2
O1—Zn1—O7	93.60 (14)	H9C—O9—H9'C	87.0
O4^i^—Zn1—O7	93.97 (14)	H9'A—O9—H9'C	70.2
O3—Zn1—O5	89.61 (13)	H8A—O8'—H8'A	115.6
O1—Zn1—O5	77.90 (12)	H8'A—O8'—H8'D	110.0
O4^i^—Zn1—O5	96.51 (12)	H9C—O9'—H9'C	116.2
O7—Zn1—O5	167.53 (13)	H9'A—O9'—H9'C	110.0
O3—Zn1—O6	75.68 (11)	O2—C1—O1	123.2 (4)
O1—Zn1—O6	90.55 (12)	O2—C1—C2	117.8 (4)
O4^i^—Zn1—O6	168.07 (12)	O1—C1—C2	119.0 (4)
O7—Zn1—O6	90.00 (14)	O5—C2—C1	110.0 (4)
O5—Zn1—O6	81.09 (11)	O5—C2—C2^iii^	109.4 (3)
C1—O1—Zn1	119.1 (3)	C1—C2—C2^iii^	110.6 (5)
C4—O3—Zn1	122.3 (3)	O5—C2—H2	109.0
C4—O4—Zn1^ii^	127.5 (3)	C1—C2—H2	109.0
C2—O5—Zn1	112.7 (3)	C2^iii^—C2—H2	109.0
C2—O5—H5	123.7	O6—C3—C4	109.7 (4)
Zn1—O5—H5	123.7	O6—C3—C3^iii^	109.8 (3)
C3—O6—Zn1	112.2 (3)	C4—C3—C3^iii^	111.1 (5)
C3—O6—H6	123.9	O6—C3—H3	108.7
Zn1—O6—H6	123.9	C4—C3—H3	108.7
Zn1—O7—H7B	111.1	C3^iii^—C3—H3	108.7
Zn1—O7—H7C	110.8	O4—C4—O3	124.9 (4)
H7B—O7—H7C	109.2	O4—C4—C3	115.8 (4)
H8A—O8—H8D	110.0	O3—C4—C3	119.3 (4)

Symmetry codes: (i) −*x*+1/2, *y*−1/2, −*z*; (ii) −*x*+1/2, *y*+1/2, −*z*; (iii) −*x*, *y*, −*z*.

### Hydrogen-bond geometry (Å, °)

**Table:**

*D*—H···*A*	*D*—H	H···*A*	*D*···*A*	*D*—H···*A*
O8—H8D···O2^iv^	0.85	2.23	3.074 (10)	174
O9—H9A···O1^iv^	0.85	2.41	2.849 (7)	113
O9—H9A···O7^iv^	0.85	2.38	3.094 (8)	142
O9—H9C···O9^v^	0.85	1.95	2.421 (14)	113
O8—H8A···O8'^v^	0.85	2.16	2.791 (12)	131
O9—H9C···O9'^v^	0.85	2.00	2.761 (10)	149
O9'—H9'C···O1^iv^	0.85	1.92	2.765 (7)	177
O9'—H9'C···O2^iv^	0.85	2.66	3.225 (8)	125

Symmetry codes: (iv) −*x*+1/2, *y*+1/2, −*z*+1; (v) −*x*, *y*, −*z*+1.
